# A Short Review on Advances in Early Diagnosis and Treatment of Ischemic Stroke

**DOI:** 10.31661/gmj.v12i.2993

**Published:** 2023-08-21

**Authors:** Bin Sun, Zhigang Wang

**Affiliations:** ^1^ Department of Neurosurgery, Qilu Hospital (Qingdao), Cheeloo College of Medicine, Shandong University, Qingdao, Shandong 266035, China

**Keywords:** Ischemic Stroke, Brain Ischemia, Artificial Intelligence, Computed Tomography Angiography, intravenous Thrombolysis

## Abstract

Ischemic stroke is a leading cause of morbidity and mortality worldwide, necessitating advancements in early diagnosis and treatment modalities. This review aims to provide an overview of recent advances in the early diagnosis and treatment of ischemic stroke, highlighting the importance of the potential impact on patient outcomes. Recent advancements have focused on various aspects of stroke care, including imaging techniques, laboratory testing, telemedicine and mobile technology, intravenous thrombolysis, mechanical thrombectomy, and collaborative systems. Advances in imaging techniques have played a pivotal role in the early diagnosis of ischemic stroke. Computed tomography perfusion imaging, advanced magnetic resonance imaging (MRI) techniques, multimodal imaging, and automated image processing tools have greatly improved the ability to assess the extent of ischemic injury. Laboratory testing has seen significant progress in identifying biomarkers associated with ischemic stroke. High-sensitivity cardiac troponin assays have improved our understanding of the cardiac component of stroke. Additionally, biomarkers such as S100B, glial fibrillary acidic protein, and neuron-specific enolase have shown promise in assessing stroke severity and prognosis. Mobile applications and wearable devices facilitate stroke symptom recognition, risk assessment, and prompt medical attention. The development of tenecteplase, a modified form of tissue plasminogen activator, has enhanced clot-dissolving efficacy. Collaborative systems, including regional stroke systems of care and telestroke networks, have optimized communication and coordination among healthcare providers. Interoperable electronic health records streamline information exchange and facilitate prompt decision-making. Mobile communication technologies enhance real-time collaboration, involving all stakeholders in stroke care. Future directions focus on artificial intelligence and machine learning algorithms for stroke diagnosis and risk assessment. Wearable devices and remote monitoring may enable continuous monitoring of stroke-related indicators. Overall, advances in early diagnosis and treatment of ischemic stroke can enhance stroke care, reduce treatment delays, and improve patient outcomes.

## Introduction

Ischemic stroke is a devastating condition that affects millions of individuals worldwide, causing significant morbidity and mortality [1]. It occurs due to the obstruction of blood flow to the brain, leading to the deprivation of oxygen and nutrients and resulting in brain tissue damage [2]. Timely diagnosis and treatment play a critical role in improved patient outcomes, as early intervention can prevent long-term disability and minimize the risk of complications [3].

Over the years, there have been remarkable advancements in the early diagnosis and treatment of ischemic stroke [4]. These advancements have revolutionized the medical landscape, providing physicians with innovative tools and therapeutic strategies that enable faster and more precise interventions [5]. This study aims to provide a comprehensive review of the recent advances in the early diagnosis and treatment of ischemic stroke, the importance of timely intervention, and its potential impacts on patient outcomes.

1.Early Diagnosis

1.1. Clinical Assessment

Clinical assessment is an essential component of the early diagnosis of ischemic stroke as it serves as the initial point of evaluation for healthcare professionals [5]. It involves a comprehensive assessment of the patient’s medical history, risk factors, and physical examination, which collectively aid in identifying potential cases of stroke promptly [6][7].

The medical history assessment collects information about the patient’s past medical conditions, such as hypertension, diabetes, cardiovascular diseases, and previous ischemic stroke or transient ischemic attacks [8]. These factors provide crucial insights into the patient’s underlying health status and the presence of potential stroke risk factors [9].

Also, risk factor assessment is an integral part of clinical assessment for early diagnosis [10]. Risk factors contributing to ischemic stroke include hypertension, hyperlipidemia, smoking, obesity, atrial fibrillation, sedentary lifestyle, and a family history of stroke [8][11]. Evaluating the presence and severity of these risk factors helps clinicians assess the patient’s susceptibility to stroke and determine the best course of action for diagnosis and treatment [12].

Physical examination during clinical assessment consists of the evaluation of vital signs, focusing on blood pressure (BP), heart rate, temperature, and oxygen saturation [13]. A sudden increase in BP or an irregular heart rhythm may indicate an acute ischemic event [14]. Moreover, a detailed neurological examination helps assess motor function, speech, and sensory abilities, which can provide valuable information about the location and extent of the stroke [15][16].

Specific examination techniques, such as assessing the National Institutes of Health Stroke Scale (NIHSS) score, aid healthcare providers in quantifying the severity of the patient’s clinical presentation [17]. The NIHSS score encompasses various neurological assessments, including motor and sensory function, language abilities, level of consciousness, and visual field testing [17][18]. This standardized scoring system assists in gauging the severity of the stroke, tracking disease progression, and determining appropriate treatment interventions [19].

1.2. Imaging Techniques

1.2.1. Traditional Non-Contrast Computed Tomography (CT)

The non-contrast CT scans allow clinicians to visualize the brain anatomy and identify areas of ischemic damage without the need for contrast agents [20][21]. Traditional non-contrast CT is a widely used imaging modality in the early diagnosis of ischemic stroke [20]. It provides fast and readily available information about the presence of acute infarction or other intracranial abnormalities that may contribute to stroke symptoms [21][22]. Indeed, non-contrast CT imaging is particularly valuable in the early stages of stroke assessment when time is critical for treatment decisions [23].

Moreover, it is a highly sensitive imaging technique in detecting hemorrhages, such as intracerebral and subarachnoid hemorrhage, which may mimic stroke symptoms but require different treatment approaches [24]. By ruling out hemorrhagic stroke, non-contrast CT helps guide appropriate management and interventions for ischemic stroke patients [25]. Early signs of ischemic stroke on non-contrast CT include hypodense areas or decreased attenuation within the brain parenchyma, representing reduced blood flow [26]. Also, such findings include loss of gray-white matter differentiation, hypo attenuation in the middle cerebral artery territory, or subtle sulcal effacement could be observed [26][27]. Combining these findings with the patient’s clinical presentation and examination could confirm the diagnosis of ischemic stroke.

In addition, non-contrast CT has additional benefits in assessing stroke-related complications and determining eligibility for specific therapies [28]. It can identify other important findings, such as large vessel occlusions, which may be suitable for endovascular thrombectomy procedures [29]. Additionally, non-contrast CT provides vital information about the extent and location of infarction, which predicts the potential impact on patient outcomes [29].

Despite its advantages, non-contrast CT does have limitations in the early diagnosis of ischemic stroke. It may not detect subtle or small infarcts in the hyperacute phase, and early imaging changes may not always be evident immediately following symptom onset [26][30]. Therefore, in the cases where clinical suspicion of stroke remains high despite a negative non-contrast CT, additional imaging such as advanced magnetic resonance imaging (MRI) techniques include magnetic resonance angiography (MRA), diffusion-weighted imaging (DWI), or perfusion-weighted imaging (PWI) may be warranted [31][32].

1.2.2. CT Angiography (CTA)

CTA is an advanced imaging technique that plays a crucial role in the early diagnosis of ischemic stroke. It combines CT scanning with the injection of a contrast agent to visualize detailed images of the arterial anatomy, allowing for the identification of occlusions or stenosis that may contribute to ischemic stroke [33][34]. Indeed, CTA scans can identify stenosis in major cerebral arteries such as the middle cerebral artery or internal carotid artery [35]. Hence, one of the key advantages of CTA is its ability to rapidly provide high-quality images of the blood vessels without the need for invasive procedures [33][36]. Also, this information is vital for determining the most appropriate treatment approach, whether it be medical management, thrombolysis, or mechanical thrombectomy [37]. CTA also facilitates the identification of collateral blood flow patterns. In cases where the primary artery is occluded, collateral vessels may offer an alternative route for blood supply to the affected brain region [38]. Assessing collateral circulation through CTA can help predict the potential for successful revascularization procedures and guide treatment decisions accordingly [39]. Moreover, CTA is particularly valuable in the hyperacute stage of stroke, where time is of the essence [39].

1.2.3. MRI

MRI is a valuable imaging modality for the early diagnosis of ischemic stroke [40]. Indeed, it could provide detailed images of the brain, allowing for the visualization of ischemic damage, identification of stroke etiology, and assessment of perfusion and diffusion abnormalities [41].

The DWI is a specific MRI sequence that is highly sensitive to changes in water diffusion in brain tissue [42]. Restricted diffusion occurs in the affected area during the early phase of the ischemic stroke process [42][43]. These findings can appear within minutes to a few hours after symptom onset, making DWI particularly useful in the hyperacute phase of stroke evaluation [42][43][44].

Also, PWI is another important MRI technique in stroke diagnosis. PWI provides information about cerebral blood flow dynamics, allowing clinicians to assess the viability of brain tissue and detect alterations in regional perfusion [45]. In ischemic stroke, regions with reduced blood flow, known as hypoperfused areas, can be visualized on PWI [46]. Coupled with DWI, PWI could identify the ischemic core (irreversibly damaged tissue) and the surrounding ischemic penumbra (potentially salvageable tissue), which is crucial in determining treatment strategies [47].

In addition to DWI and PWI, MRA can be applied in the early diagnosis of ischemic stroke by assessing the blood vessels’ integrity and identifying stenosis or occlusions [48]. So, information from MRA is vital in determining the underlying cause of the ischemic stroke, such as atherosclerosis or embolism [49]. Hence, the advantage of MRI over other imaging modalities is its superior soft tissue contrast and the ability to obtain multiplanar views of the brain [41][50]. Also, it allows for more detailed evaluation of the extent and location of ischemic infarction, helping guide treatment decisions, and is beneficial in differentiating ischemic stroke from other conditions, such as intracranial hemorrhage or tumors, which may have similar clinical presentations [51][52].

1.3. Laboratory Testing

Advancements in laboratory testing have played a significant role in the early diagnosis of ischemic stroke, leading to more timely and accurate interventions [53]. Recent advancements in laboratory techniques and biomarkers have improved our understanding of stroke pathology and provided valuable tools for early diagnosis [54]. One important advancement is the use of high-sensitivity cardiac troponin assays [55].

Cardiac troponin is a biomarker released into the bloodstream during cardiac injury, including myocardial infarction [55]. Research has shown that elevated levels of cardiac troponin can also be detected in patients with ischemic brain stroke, indicating a potential cardiac component or coexisting cardiac injury [54]. High-sensitivity assays enable the detection of very low levels of cardiac troponin, allowing for the identification of subtle myocardial injury that may have contributed to the stroke event [55].

In addition to cardiac markers, several other biomarkers have shown promise in the early diagnosis of ischemic brain stroke. For instance, S100B, a protein released by damaged brain cells, has been found to be elevated in stroke patients [56]. Measuring S100B levels in the bloodstream can help determine the severity and prognosis of the stroke [57]. Other biomarkers, such as glial fibrillary acidic protein [58] and neuron-specific enolase [59] shown potential as indicators of stroke severity, brain injury, and prognosis.

Other biomarkers, such as matrix metalloproteinase (MMPs) [60], inflammatory markers like C-reactive protein [61], and interleukin-6 [62] have shown promise in early stroke diagnosis. MMPs are enzymes involved in extracellular matrix remodeling, and their dysregulation is implicated in the pathogenesis of stroke [60]. Elevated levels of MMPs in the blood have been linked to an increased risk of stroke and can serve as valuable markers for early diagnosis [63]. Complete blood count and coagulation profile tests are routinely performed in patients with suspected ischemic stroke [64]. These tests evaluate parameters such as platelet count, hematocrit, and international normalized ratio, providing information about the patient’s coagulation status and the potential risk of bleeding or hypercoagulable states [65].

Furthermore, laboratory testing may include lipid profiles to measure cholesterol and triglycerides, as dyslipidemia is a known risk factor for ischemic stroke [66]. Assessing blood glucose levels is also essential, as hyperglycemia is associated with poorer outcomes and increased stroke severity [67]. A complete evaluation of other metabolic parameters, such as renal function, electrolyte abnormalities, and liver function can determine the overall health status of the patient and guide treatment decisions [68].

Advancements in genetic testing have also provided valuable insights into the risk and etiology of ischemic brain stroke [69]. Genetic testing can identify specific gene mutations, such as those related to coagulation disorders (e.g., Factor V Leiden mutation), that increase the risk of stroke [70]. Detecting these genetic markers early on allows for targeted interventions and preventive measures in individuals at high risk of stroke [71]. Genetic testing can also identify genetic variants associated with specific stroke subtypes, allowing for tailored treatment approaches [72].

Moreover, the introduction of point-of-care testing (POCT) devices has revolutionized laboratory testing in stroke diagnosis [73]. These portable devices provide rapid and accurate results at the bedside, enabling healthcare providers to evaluate biomarkers such as glucose levels, coagulation profiles, and lipid profiles almost immediately [74]. POCT allows for timely decision-making, especially in time-sensitive scenarios such as thrombolytic therapy, where prompt assessment of coagulation status is crucial [73][75].

1.4. Telemedicine and Mobile Technology

Advancements in telemedicine and mobile technology have revolutionized the early diagnosis of ischemic stroke, particularly in remote or underserved areas [76]. These technological innovations have facilitated timely assessment and intervention for stroke patients, ultimately improving outcomes [77].

Telestroke programs could provide video conferencing and real-time communication to connect stroke specialists with healthcare providers in remote locations [78]. Through telemedicine, stroke experts can remotely assess patients, review imaging studies, and provide immediate guidance to local healthcare teams [79]. This enables rapid evaluation and early diagnosis of ischemic stroke, ensuring patients receive timely treatment, such as thrombolysis or mechanical thrombectomy [80].

These applications use validated algorithms and guidelines to assess symptoms, calculate stroke risk, and provide necessary recommendations for seeking medical attention promptly [81].

Also, mobile technology allows for the transmission of neurological examination findings, vital signs, and imaging studies in real-time, facilitating fast and accurate decision-making by stroke specialists [81]. For example, mobile applications can securely send CT scans to stroke experts for immediate interpretation, aiding in promptly diagnosing and determining appropriate treatment strategies [82].

Furthermore, telemedicine and mobile technologies have enhanced stroke education and awareness. Online platforms and mobile applications provide educational resources, raising public awareness about stroke risk factors, signs, and symptoms [83]. Increasing knowledge about stroke allows individuals to recognize stroke symptoms promptly and seek medical help without delay, resulting in earlier diagnosis and reduced time to treatment.

2. Early Treatment

2.1. Intravenous Thrombolysis

Advancements in intravenous thrombolysis have greatly improved the early treatment of ischemic stroke, allowing for more effective and timely interventions [84]. Intravenous thrombolysis involves the administration of an anti-coagulant medication, typically a tissue plasminogen activator (tPA), to dissolve the blood clot causing the stroke [85].

One significant advancement in intravenous thrombolysis is the extension of the treatment window [86]. Previously, intravenous thrombolysis was only recommended within a limited time frame, typically within 4.5 hours after symptom onset [87]. However, recent studies have shown that selected patients may benefit from thrombolysis beyond this time window, up to nine hours or even longer in some instances [88][89]. This extended treatment window has expanded the number of eligible patients who can receive timely thrombolytic therapy and improved their chances of functional recovery [88].

Moreover, advancements in neuroimaging have facilitated the identification of patients who may benefit from thrombolysis despite initially presenting with uncertain symptom onset [89]. Imaging techniques like perfusion imaging and mismatch analysis can assess the extent of ischemic penumbra and the presence of large vessel occlusion, contributing to treatment decision-making [90]. These advances have allowed more patients with ischemic stroke to receive thrombolysis, even when the exact time of symptom onset is unknown [90].

Another significant advancement in thrombolytic therapy is the development of tenecteplase, a modified form of tPA that has several advantages [91]. Tenecteplase has a longer half-life, allowing for a single bolus administration instead of the continuous infusion required with tPA. It has increased fibrin specificity, which makes it more potent and effective in dissolving clots [91][92]. Previous studies [92][93] demonstrated improved reperfusion rates and functional outcomes compared to standard tPA.

Furthermore, advancements in pre-hospital management and stroke care systems have contributed to the early treatment of ischemic stroke with thrombolysis [94]. Mobile stroke units, equipped with neuroimaging and tPA administration capabilities, bring stroke expertise and interventions directly to the patient’s location [95]. These units allow for early triage and treatment initiation, reducing the time from symptom onset to thrombolysis and improving patient outcomes [96]. These advancements have allowed a broad range of eligible patients to receive thrombolysis, even in cases of uncertain symptom onset, leading to enhanced reperfusion, improved functional outcomes, and reduced disability rates for individuals with ischemic stroke [97].

2.2. Mechanical Thrombectomy

Advancements in mechanical thrombectomy have revolutionized the early treatment of ischemic stroke, providing a highly effective and minimally invasive intervention for patients with large vessel occlusions [98]. Mechanical thrombectomy involves using specialized devices to remove or disrupt the blood clot causing the stroke physically [99].

One significant advancement is the development of stent retrievers, which have greatly improved the success rates of mechanical thrombectomy [100]. Stent retrievers have demonstrated excellent recanalization rates and higher chances of functional independence compared to traditional methods of clot removal, leading to improved outcomes for patients with ischemic stroke [101][102].

Another notable advancement in mechanical thrombectomy is the expansion of the treatment window [103]. Previously, mechanical thrombectomy was primarily recommended within six hours of symptom onset [103]. However, clinical trials have demonstrated the efficacy of mechanical thrombectomy for up to 24 hours in carefully selected patients with ischemic penumbra, offering a longer time window for intervention and expanding the number of eligible candidates [104][105].

Also, advancements in device technology have played an important role in improving mechanical thrombectomy outcomes [106]. Smaller, more flexible, and navigable catheters have been developed, enabling better access to the occluded vessel and enhancing the procedural success rate [107]. Newer-generation thrombectomy devices, such as aspiration catheters and stent retrievers with improved design and coatings, provide efficient clot removal while minimizing the risk of distal embolization and vessel injury [108][109].

3. Collaborative Systems

Advancements in collaborative systems have significantly improved the early treatment of ischemic stroke by enhancing communication and coordination among healthcare providers, stroke specialists, and emergency response teams [110]. Collaborative systems bring together various stakeholders involved in stroke care to easier the process and expedite treatment delivery [111].

One key advancement is the implementation of regional stroke systems of care. These systems establish networks among hospitals, emergency medical services, and stroke centers to ensure coordination and rapid response to stroke emergencies [112]. Regional stroke systems facilitate the identification of patients with stroke in the pre-hospital setting and provide a simple pathway for their referral to comprehensive stroke centers or specialized stroke units capable of delivering timely and appropriate interventions [113].

Through telemedicine technology, stroke experts can evaluate patients, review imaging studies, and guide treatment decisions [81]. Hence, healthcare providers in remote or rural regions could promptly access expert advice, ensuring the early initiation of appropriate treatments [83].

Advancements in information sharing and electronic health records (EHRs) have also played an important role in collaborative systems for early stroke treatment [114]. Interoperable EHRs allow for the transfer of patient information, including medical history, imaging studies, and laboratory test results [115].

4. Future Directions

Future directions for the early diagnosis and treatment of ischemic stroke are focused on advancing technology, improving risk assessment methods, and enhancing treatment options [116]. These directions can potentially optimize stroke care and improve patient outcomes.

One important future direction is the development and integration of artificial intelligence (AI) and machine learning algorithms into stroke diagnosis and risk assessment [117]. AI algorithms can analyze complex datasets, such as imaging studies and patient data, to detect subtle signs of stroke or identify high-risk individuals [118]. Hence, it enhances the accuracy and efficiency of stroke diagnosis, allowing for earlier intervention and treatment.

Additionally, the integration of wearable devices and remote monitoring technologies may enable continuous monitoring of stroke-related biomarkers or physiological parameters [119]. These devices can detect early changes in key indicators, such as BP, heart rate, or glucose levels, allowing for early identification of stroke symptoms and prompt medical intervention [120]. Real-time monitoring can provide critical information to healthcare providers, facilitating timely diagnosis and treatment decisions [121].

Another future direction is the advancement of targeted therapies for stroke treatment. Currently, reperfusion therapy, including thrombolysis and mechanical thrombectomy, is the mainstay of treatment for ischemic stroke [122]. However, the development of new pharmacological agents that target specific pathophysiological pathways, such as neuroinflammation or excitotoxicity, may enhance neuroprotection and improve outcomes in ischemic stroke [123][124]. These targeted therapies may combine with reperfusion strategies or standalone treatments to minimize brain damage and maximize recovery [125].

Future directions also emphasize the importance of stroke prevention through public health initiatives, education, and lifestyle interventions [126]. Promoting awareness about stroke risk factors, encouraging healthy lifestyle behaviors, and implementing effective screening programs can help identify high-risk individuals and allow early intervention to prevent stroke occurrence or recurrence [127].

## Conclusion

Advances in the early diagnosis and treatment of ischemic stroke have significantly transformed stroke care, leading to improved clinical outcomes. Early diagnosis facilitated by innovative imaging techniques and prompt intervention through thrombolytic therapy and mechanical thrombectomy has enhanced recovery rates and reduced disability. Furthermore, the implementation of collaborative systems, such as telemedicine, provides access to specialized stroke care in remote areas. Continued innovation and research in stroke management could optimize patient care and outcomes in the future.

## Conflict of Interest

None.
